# Strategies to reduce visual attention changes while learning and training in extended reality environments

**DOI:** 10.1007/s12008-022-01092-9

**Published:** 2022-12-11

**Authors:** Luis Bautista, Fernanda Maradei, Gabriel Pedraza

**Affiliations:** Universidad Industrial de Santander, Bucarmanga, Colombia

**Keywords:** Augmented reality, Virtual reality, Mixed reality, Attention, Graphic user interface

## Abstract

Computer-Based training (CBT) is a widely used strategy in interactive learning and skills training to provide the trainee with information while in training. Extended Reality (XR) is a set of technologies used in the fourth industrial revolution for this purpose. However, an important XR limitation is the cognitive overload in trainees due to continuous changes in attention. The effects of the changes in attention on cognitive load have been studied in environments such as printed material and desktop PC’s. However, such effects are not yet fully known on XR platforms. This study’s aim was to identify strategies to reduce the changes in attention in instructional/educational materials, which use extended reality to present information to trainees. The presented information can guide the use of combined strategies to reduce the cognitive overload generated by XR display platforms. Therefore, an extensive literature review was carried out. From a set of 1946 articles, 53 studies were selected. The selected studies evaluated the cognitive load in instructional materials that used XR as a visualization platform. The results showed three strategy groups: those associated with the spatial integration of information, those associated with the use of visual features and those associated with the content segmentation. Effects of this strategies in the user performance and user cognitive load are discussed.

## Introduction

Extended Reality (XR) is considered a set of technologies including virtual reality (VR), mixed reality (MR) and augmented reality (AR), ALL part of the fourth industrial revolution and are widely used in learning and training environments using the computer-based paradigm. For example, augmented reality is widely used to allow combining objects (real and virtual) and providing spatially positioned information in training environments [1, 2]. According to several studies, the main utility of AR is to provide procedural and support information during training [3–7]. According to Lebel [7] y Gavish [4], the information provided improves trainee performance and it´s a fundamental component while training since it provides initial support for the learning and automation phases [8]. The information is delivered to the trainee using a Graphical User Interface (GUI), through small units called "Displays" [9]. However, according to Evans, [10] y Acampora [11] AR advantages in training could be reduced by cognitive overload generated by the poor disposition of information. An inadequate disposition of information overloads the trainees working memory and reduces cognitive resources available for learning [12, 13]. As established by the cognitive load theory (CLT) [14], working memory overload can have an external source and increases the cognitive resources consumption due to the attention changes required to search for specific information within the virtual environment [15]. Therefore, Ayres and Sweller [16, 17], state that the Split attention principle is important to avoid attention changes. This principle states that, trainees should avoid dividing visual attention among multiple sources of information to ensure a proper learning process. Split attention of instruction occurs when trainees are required to divide their visual attention and mentally integrate information from separate (spatial or temporal) sources. Because each information source is essential to understanding the displayed instructional material, the external cognitive load is likely to be increased. Applying the principle has challenges: the presentation format selection [16, 18] and the positioning of information in allotted virtual spaces [19, 20]. On the other hand, other principles such as signaling [21], and coding, are applied to drive attention during learning, but the information found is segmented or dispersed. Despite the wide use of extended reality, few studies have evaluated its impact on cognitive load during multimedia learning [22]. *Akçayır* [23] mentions the lack of data on cognitive load factors in said studies associated with ER technologies and the existing ones**,** show contradictory results. Similarly, *Ens* [24] mentions the need to study the effect on cognitive load and trainee performance, when multiple information spaces are used in augmented reality. Also, Garcia-San Juan [25] states that the effect of separation and presentation of information on multiple displays on the trainee performance is unknown. Finally, Rashid [26] shows the need to study split attention and information distribution (in the virtual environment), to reduce changes in the trainee’s visual attention. For this reason, the work aim was to review the literature to identify strategies oriented to avoid changes in attention while training with multimedia and XR technologies. Attention changes increase cognitive load by this, in this study several techniques used to deal with changes in attention in Augmented reality, Virtual Reality and Mixed Reality were reported.

In this review, three groups of strategies were identified: Regarding to spatial integration, regarding to visual attributes uses and regarding to content segmentation. Effects of spatial integration formats and contiguity effect condition was identified. Empirical studies were identified that relate visual attributes such as color and signaling forms, with negative effects on the trainee performance, the attention changes and distraction [1, 2, 27, 28]. Content segmentation techniques used to reduce cognitive load and improve the user performance in training and learning material was identified. Finally, results are discussed and the few studies that evaluate cognitive load due to the graphical user interface configuration in extended reality technologies are evidenced. The results of this study may serve as a guide for graphical user interfaces design for: instructional, learning and training materials with extended reality technologies, which reduce attention changes and cognitive overload in trainees. Despite this, the authors consider it necessary to carry out more in-depth studies that relate the properties of the user interface with the effect on changes in attention and cognitive load.

## Theorethical framework

### Training with interactive technologies

In recent years, extended reality technologies have been emerging within the fourth industrial revolution as tools for simulation-based training (SBT) [29]. The impact identified is relevant, especially in industrial and medical applications [30]. According to *Shaywitz* [31], a simulation is "a recreation of reality aspects in a setting or environment". Interactive simulators are used as an effective tool for KSA (Knowledge, Skills, and Attitude) training, incorporating several levels of realism: physical, functional, or psychological [31]. Knowledge is obtained by an information acquisition and subsequent learning; Skill is developed through repetition and regular practice; finally, Attitude, relates the knowledge integration and the perform the task ability. SBT is used to create training environments related to high-risk situations [32] without compromising the trainee integrity and safety. Additionally, SBT can represent out or the ordinary and unknown environments with 24/7 availability for practice [31]. However, as *Vandewaetere* states, the training effectiveness using SBT depends on an adequate instructional design that includes authentic and real learning tasks in a simulated environment [9].

### Extended reality in learning and training

**Fig. 1:**
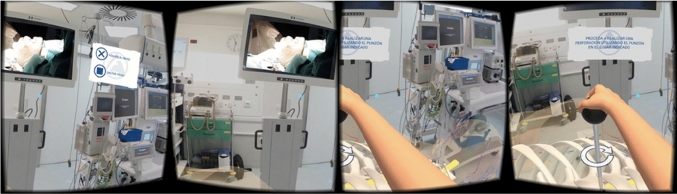
VR Application designed for operating room (OR) equipment use training [37]

Extended reality is an umbrella term that covers VR, AR and MR. XR tech modifies the user interface to immerse the user in the virtual environment (VR) or augments the user´s surrounding (AR), or both of those (MR). Virtual reality (VR) has made a breakthrough and is now used in a wide range of applications. It allows a user to be completely immersed and involved, making them feel "present"[33]. VR has been widely used for training applications due to the ability to design specific, complex and safe environments at low cost. In fact, training using virtual reality can achieve performance levels similar to real training environments [34], and in some cases, performance has improved [35, 36]. For instance, in educational, industrial and medical applications, VR has shown wide efficacy as a training tool. In medical applications, such as surgical equipment training in the operating room [37], González concluded that the automation of procedural steps was the main contribution. Additionally, the trainee can remember the most important steps thanks to the possibility of repeating the procedure as many times as necessary. *Siu* [38] proposed a VR-based system to predict the acquisition of surgical skills. VR improved the skill level in basic surgical tasks such as: Peg transfer and Needle passing tasks, thanks to the adaptive VR incorporation. The main achievements were improvement in the ability to interactively assess learning and skills impairment, optimize skills re-learning and surgical skills practice. *Sattar* [39] evaluated VR in abdominal surgery training using laparoscopy with 87 medical students, compared to video and text-based learning. *Sattar* found that motivation and knowledge transfer was significantly higher for VR training compared to other methods. Also, it was identified that VR fostered active learning. Mickiewicz [40] evaluated the VR impact on otolaryngology resident’s training. VR training allowed the participants to significantly improve virtual antromastoidectomy performance and resulted in a shortening surgery duration and number of mistakes made. Also, VR training provides a structured, safe and supportive environment to familiarize oneself with complex anatomy and to practice surgical skills. *Ho* [41] used virtual reality for training in medical device’s assembly, finding superiority in VR training compared to traditional training. *Ho* found that incorporating AI with VR achieved higher effectiveness scores, increased confidence level and shorter training time compared to traditional training. Besides, VR has been used to evaluate the competencies of residents in orthopedics [42], as a remote training tool in the Covid-19 pandemic [43], introducing curriculum changes to incorporate VR [42]. In other applications, *Pila* [44] uses VR in training systems for inspection and verification tasks on production plants. VR increased the realism level, immersion and interaction between the components, the environment and the user. *Kalkan* [45] implemented VR to improve the performance in complex assembly task**s**. Results revelated that VR-based training reduced training time up to 25% per subject and increased performance up to 27%. *Lee* [46] developed a VR application for procedural tasks training in factories, resulting in performance improvement. However, according to *Le*, multiple design considerations need to be taken into account in designing VR (GUI) interfaces for factory training (Fig. 1).

**Fig. 2:**
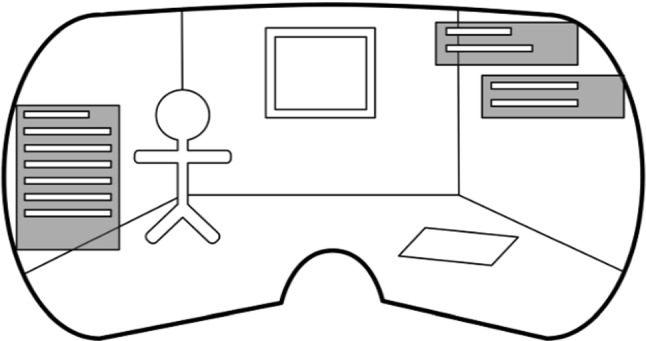
Augmented reality. Everything you see is real, with an additional layer of superimposed data in your field of view. O´Conell. [51]

Another XR technology is Augmented Reality. AR is an interactive technology that combines digital and physical information through different technological devices interacting with the surroundings/environment in real-time [47], or as *De la Torre* mentions [48], *“it is a technology that allows user interaction with the physical and real-world that surrounds it"*. AR combines computer-generated 3D objects and text superimposed on real images and video, in real-time, as seen in Fig. 2. Therefore, AR is a technology that facilitates the integration of real elements with added virtual ones, to create a new communicative scenography [49]. *Santos* [50] states three features that make AR an interactive technology to create learning experiences: *Added information* in the real world improve perception by superimposing real objects and virtual text, this reduces the cognitive load due to reduced working memory usage; *Contextual visualization* improves spatial integration by providing relevant clues in the real environment that help the trainee to build his/her knowledge base; finally, the simultaneous use of *vision and touch* senses to present information improves integration based on body movement.

Often, AR is used in initial phases of training with remote assistance for maintenance tasks. For instance, AR is widely used in medical procedures to increase accuracy [52] and several applications used AR in surgical skills training [53, 54]. *Martín-Gutierrez* [55] used AR to train engineering students in spatial skills. The experimental group improved their spatial skills significantly compared to the control group. Similarly, *Lee* [56] shows AR applications for military vehicle and equipment maintenance training. In manufacturing industry, it is used for vehicle assembly line training, electronic devices assembly [57] and industrial plants inspection. *Gavish* [58] compared AR with other maintenance and assembly training methods. In the evaluation, operators who used AR had significantly better performance in the task-time compared to those who did not. *Westerfield* [27], combined AR with an intelligent tutoring system for computer motherboard assembly training, and the results showed an increase in user scores and performance. In medical applications, AR has been used in surgical procedure training. *Sanne* [59], analyzed four AR simulators for laparoscopic surgery training and shows the advantages for training basic skills such as manual coordination, grip and traction, as well as for advanced skills, such as clip application, suture placement, dissections, among other. Likewise, the importance of AR to provide information during the procedure is evidenced, mainly in the early stages of training. Finally, *Chowriappa* [60], demonstrated how AR improves skills acquisition with minimum demand for cognitive load in the training of Uretrovesical Anastomosis—UVA. This study emphasizes that AR allows the anatomical and scenario variability, didactic education and user´s adaptation, in an integrated way along with the true practical acquisition of surgical skills.

Mixed reality is a technology in which virtual objects and other digital information are superimposed over the real-time view of the physical objects providing a composite view with guides for carrying out a task with or on physical objects. For instance, Kobayashi [62] implemented a mixed reality prototype to transfer knowledge from experts’ surgeons to novice students. Using a mixed reality interface, a set of cues were projected to guide “blind insertion” invasive procedural training. Margarido [61] designed a mixed reality simulator to train pinpoint insertion of intravenous needles. Using an OST, the mixed system provides a visual guide to the insertion task that helps novice students performing the central venous catheterization procedure. The MR system development evidenced a significative difference in task time and number of errors made by novice participants. Brunzini [63] applied mixed reality to train lumbar puncture. A training scenario was developed for novice students to practice. Results confirmed the great potential of extended reality as a support tool for future medical procedures, both for medical training and when performing real life procedures. Other mixed reality applications have been used in the industrial field such as on-the-job training. Sautter [64] shows potential for effective industrial sector training with regard to key aspects of constructivist learning. In addition, MR should be applied regarding the specific learning tasks, for instance, integrating workers in new planning processes right from the beginning. In maintenance tasks, Su [65] proposes an auxiliary equipment maintenance system based on Mixed Reality technology. The system drives the maintenance procedure guidance and equipment training phase, where users only need to follow the steps set by the system. MR can help repairment personnel check and carry out maintenance operations without a maintenance manual, especially in case of difficult complex industrial equipment maintenance as well as in its potential difficult circumstances.

### Working memory and cognitive load

The cognitive factors (or cognitive ergonomics) studied by Human Factors, comprise, on one hand, communication and teamwork, and on the other hand, perception, work memory, reasoning and motor skills [66]. Long-term memory is the place where knowledge is stored, likewise, short-term memory or working memory is responsible for processing the information required during task execution. Or as Baddeley [67] defines it, Working Memory is a dedicated system that maintains and stores information in the short term, in periods between 15 and 30 s, and is the basis of human thought processes related to the conscious activity that a person develops. Based on the limited processing capacity of working memory, Sweller proposes a theory of cognitive load [68, 69] and later Mayer proposes the cognitive theory of multimedia learning [70, 71]. These theories raise premises related to the use of limited cognitive resources and the limited capacity of a learner when dealing with new information. The cognitive load that a learner presents can be a) Intrinsic, it is the load inherent to the task complexity; b) Extrinsic, is the load that saturates, pollutes, and affects working memory, generally caused by spatially misplaced instructional materials or user interfaces whose representations hinder cognitive processes; c) Germane is responsible for contributing to learning. The three types of cognitive load are complementary, so it is usually sought to reduce the Extraneous cognitive load, free up working memory resources, and achieve more efficient learning [72]. Therefore, the main strategy proposed by Sweller, to deal with cognitive overload, is the reduction of extraneous cognitive processing. For example, in Information Search and Matching [17, 68]. Based on the evidence, Sweller and Chandler [68, 73], have proposed some principles for the reduction of extraneous cognitive load: coherence, redundancy, signaling, temporal contiguity, and spatial contiguity. As Jeroen [3] explains, the Redundancy Principle indicates that the presentation of redundant information typically has a negative impact on learning. However, learners have to find out that the information from different sources is actually redundant, which is a cognitively demanding process that does not contribute to meaningful learning; the Signaling Principle or attention-focusing principle indicates that learning may be improved if the learner’s attention is focused on the critical aspects of the learning task or the presented information. It reduces the need for visual search and so frees up cognitive resources that may then be devoted to schema construction and automation, with positive effects on transfer test performance; the Temporal Contiguity principle or temporal split-attention principle indicates that learning from mutually referring information sources is facilitated if these sources are not separated from each other in time, i.e., if they are presented simultaneously, and the Spatial Contiguity or split-attention principle, refers to the finding that higher transfer test performance is reached when mutually referring information sources are physically integrated with each other in space. Finally, the spatial contiguity and signaling principles is a special interest for the spatial integration of information and attention changes, due to the ability to relate objects in a 3D space and associate them in a spatial context.

### Split attention and signaling principles

According to Ayres and Sweller [16], the split attention principle establishes that, for instructional material design, it´s important to prevent trainees from dividing their attention among multiple sources of information, to ensure mental integration. Due to the need to integrate multiple sources of information, the extraneous cognitive load increases having a negative impact on learning. This effect is observed when trainees who study with integrated information formats outperform those who study with the same information but in non-integrated formats. In Kalyuga [74], there is evidence that spatial integration of information sources reduces attention shifts without the need for additional relationships. This increases effective working memory, reducing the cognitive load, and increasing test performance. Likewise, the author mentions the need to be careful to avoid information redundancy that could eliminate any positive effect.

Mayer [71], identifies some conditions where the spatial contiguity effect is stronger. a. Trainees with low prior knowledge: b. Spatial integrated formats are more effective than separate formats for low-knowledge trainees, but not for high-knowledge trainees [75]; c. Text and non-redundant images, it is applied when multiple sources of information would be unintelligible in isolation; d. Complex Lessons are applied when the material is complex, but may not apply when the material is so simple that the separate design does not overload the cognitive system; e. Interactive formats, the principle of spatial contiguity can be strengthened when trainees create integrated presentations by moving text to relevant parts of a graphic. In the same way when the material has high levels of interactivity. In a Meta-analysis, Schroeder [76], studied some variables like: instructional medium, image type, element interactivity, learning experience, redundancy of learning material, domain, education level, world region, testing type, duration of intervention, and incentives. Finally, the analysis found some correlated effects: learning experience has a small effect, redundancy of learning material has medium effect, educational level has big effect. Pouw [77] studies in two experiments the split-attention effect. Results indicate that increased cognitive load demands due to spatial separation of information is a viable underlying mechanism for the split-attention effect. However, spatial separation is likely not the only, nor a sufficient, condition for the “split-attention effect” to occur.

According to Van Gog [78], The signaling principle, also known as the cueing principle, refers to the finding that people learn more deeply from a multimedia message when cues are added that guide attention to the relevant elements of the material or highlight the organization of the essential material. In multimedia learning, this means that the attention allocation of novices, who lack prior knowledge of a task, may rely more on the characteristics of the stimulus material. Some visual features are used to implement signaling principle, for instance, text-based cues, Pictured-based Cues, Coding cues, Cues based on animation. Several techniques are used to signaling: Color, Blinking and movement. Grogorick [79], Moon [80], Jin [81], Bernhard [82], Grogorick [83], Jamet [84], Jarodzka [85], used color coding to guide attention towards relevant information. Finally, Grogorick [83] and El-Nasr [86] used element movement to guide attention. Position changes and short paths were used.

## Materials and methods

### Procedure

#### Review question

The methodology proposed by Kitchenham [87] and Petersen [88] was used to conduct the review. This study’s aim was to review the literature to identify strategies oriented to avoid changes in spatial attention or reduce its effect as well as how these strategies are implemented within the graphical user interface to provide procedural information to trainees. Therefore, the following review question (RQ) was posed:

RQ: Which strategies are applied to reduce spatial attention changes and cognitive load in learning environments that uses Extended Reality to provide information to trainees?

#### Research context

PICOC (Population, Intervention, Comparison, and Outcomes) was used to identify keywords and create search strings to answer the review question:

- **Population**: Journals papers, book chapters, conference proceedings and doctoral thesis.
- **Intervention**: User experimental studies that evaluated: performance (time, number of errors), cognitive load and/or mental effort.
- **Comparison**: comparison but without doing any intervention
- **Results**: Set of studies that evaluates user interfaces in XR technologies, performance (times, number of errors), cognitive load and/or mental effort.
- **Context / level of coverage**: Articles in English and Spanish published since 1990.

#### Keywords

The identified keywords are shown in Table 1. A list of 12 search terms was obtained, grouped into four categories: a. Related to User's activity; b. Related to cognitive effect; c. Related to XR Technology; d. Related to user interface. Additionally, synonym terms were included. Finally, a search string was built using the Boolean connectors AND, OR and NOT.

**Table 1:** Keywords grouping

A	B	C	D
*Cognitive effect*	*User activity*	*Technology*	*Interactivity*
Cognitive load	Learning	Virtual reality	User Interface
Extraneous cognitive load	Training	Augmented reality	
Split attention	Procedural information	Mixed reality	
Selective attention			
Visual focus			

#### Scientific databases

The strings were used to search in four multidisciplinary databases: EBSCO, Web of Science, SCOPUS, with a time frame between 1990 and 2022 (Table 2).

**Table 2:** Research strings

Data base	Research string	Results (Studies quantity)
EBSCO	(“Cognitive load” OR “Extrinsic cognitive load” OR “Split Attention OR “selective Attention” OR “Visual Focus”) AND (learning OR training OR procedural information) AND (“Augmented reality” OR “Virtual Reality” OR “Mixed reality”) AND (“user interface”)	1893
Web of Science (WoS)	(TI = (“cognitive load” OR “extrinsic cognitive load” OR "Split attention" OR "selective attention" OR "visual focus" AND learning OR Training OR "procedural information" AND "augmented reality" OR "mixed reality" OR "virtual reality" AND "user interface")) AND LANGUAGE: (English, Spanish) AND DOCUMENT TYPE: (Article)	6385
SCOPUS	((TITLE-ABS-KEY (“Cognitive load” OR “Extraneous Cognitive load” OR “Split Attention” OR “Selective attention” OR “Visual focus”) AND TITLE-ABS-KEY (“procedural information” OR training OR learning) AND TITLE-ABS-KEY ("augmented reality" OR "mixed reality" OR "virtual reality") AND TITLE-ABS-KEY (“user interface”)))	5352

#### Inclusion and exclusion criteria

**Fig. 3:**
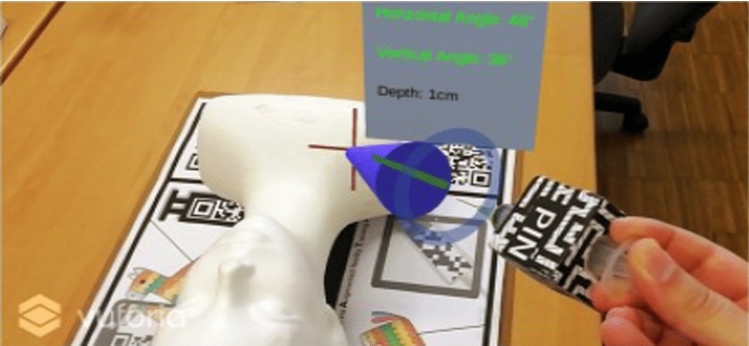
Mixed reality System development by Margarido [61]

According to Pettersen [88] suggest, To guarantee the study identification process the first author generated a validation set of studies after the second author completed the search and inclusion/exclusion process (Fig. 3).

Inclusion criteria applied:

- Journal Articles, Conference Proceedings, and Doctoral Thesis.
- Studies in Spanish or English language
- Studies that evaluated cognitive load, mental load, mental effort, performance or learning.
- Studies that evaluated instructional material oriented to learning and training.
- Studies that used as display devices: desktop PC’s, mobile devices, High definition displays, Headset displays, for Augmented, Mixed Reality, Virtual Reality and Multimedia environments.
- Studies on demographics ranging between 18 and 50 years old.

Exclusion criteria applied:

- Non-experimental studies
- Reviews and studies without users
- Studies about cultural applications
- Studies including participants with cognitive disabilities, low vision, vision problems
- Studies with underage participants or over 50 years old.

With the set of 53 chosen primary studies, a quality assessment was carried out using the following questions:

- Is the study’s aim clearly identifiable as an assessment that measures users' cognitive load or performance?
- Do the study findings relate aspects, such as spatial contiguity or visual factors**,** with measures of users' cognitive load or performance?
- Are the experimental studies clearly defined?

#### Studies selection

**Fig. 4:**
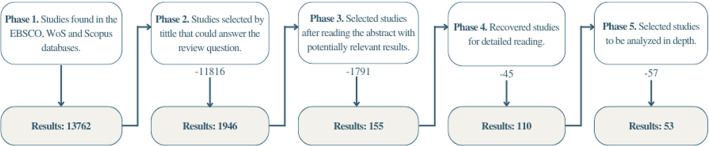
Studies selection

From database searches, 13,762 articles were obtained. Once the analysis by title was made, the following results were found (see Fig. 4):

**EBSCO**: A total of 1893 results were obtained, of which, once the title was reviewed, 492 were obtained, excluding 1401 because they did not meet the inclusion criteria. There were 49 articles selected from the abstract reading phase, also excluding 443 articles because they did not have experimental studies. When a detailed analysis was performed, 39 articles were obtained, excluding 10 for not providing relevant information for this research. Finally, 27 articles were obtained with valuable information, after exclude 12 studies by do not answer the quality assessment questions.

**WoS**: A total of 6385 articles were obtained, of which, after performing a title analysis, we obtained 975, excluding 5410 articles for not meeting the inclusion criteria. There were 57 articles retrieved from the abstract reading phase, excluding 918 for not having experimental studies. When performing a detailed analysis, 36 articles were obtained, excluding 21 for not providing relevant information for the research. Finally, there were 11 articles selected with valuable information for the study after exclude 25 studies by quality assessment questions.

**Scopus**: A total of 5484 articles were obtained, once a title analysis was performed, 479 were obtained, excluding 5005 because they did not meet the inclusion criteria. There were 49 articles selected from reading the abstracts, excluding 430 articles because they did not have experimental studies. When performing a detailed analysis, 35 articles were obtained, excluding 14 for not providing relevant information for the research. Finally, 15 articles were obtained with valuable information for the study after exclude 20 studies by quality assessment questions.

#### Classification and analysis

**Fig. 5:**
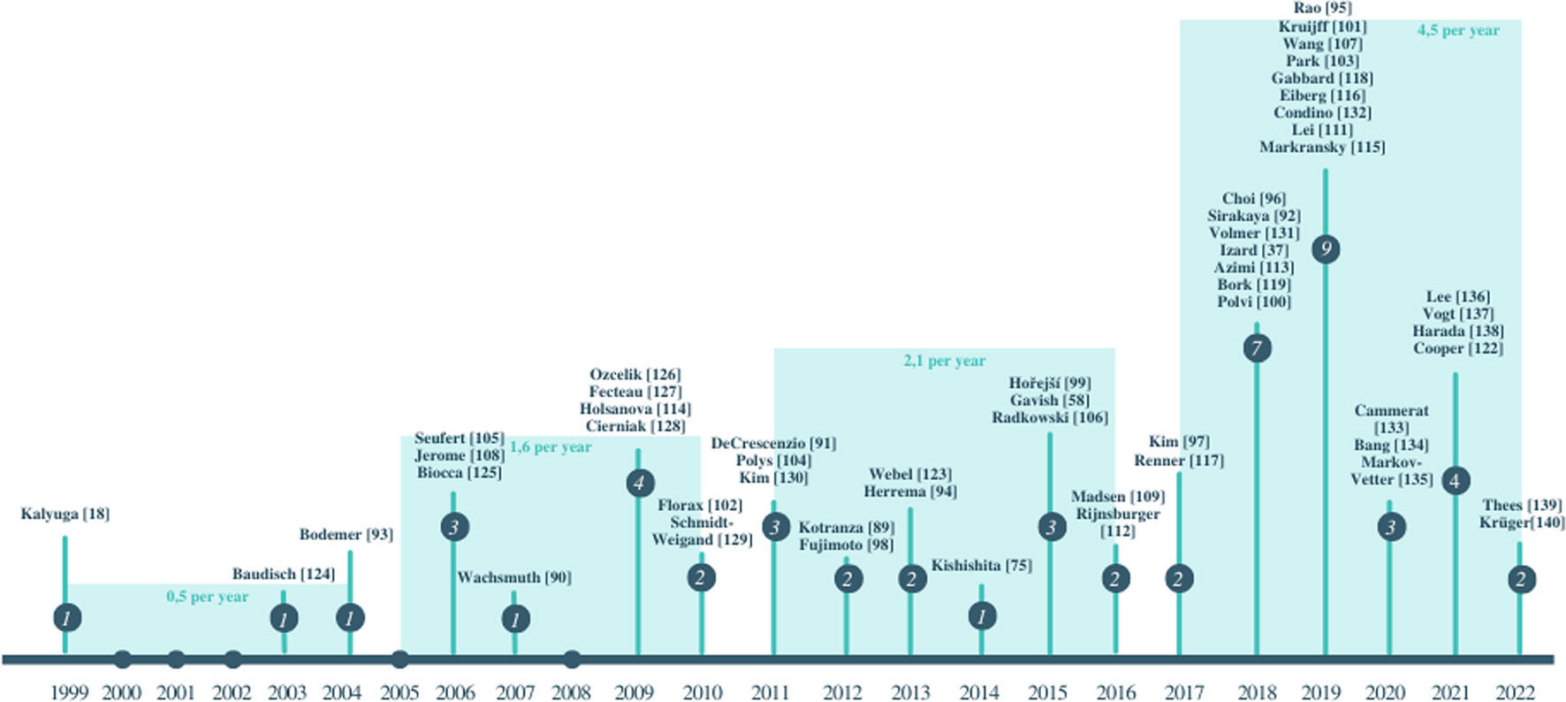
Publications per year

The strategies identified were grouped according to the factor type to avoid split attention. Afterward, the documents belonging to each topic were counted. For example, for the spatial integration strategy, the distance was identified as the main property. For the addressing strategies, characteristics related to coding, signaling, and segmentation were identified (Fig. 5).

### Evaluation validity

#### Descriptive validity

Descriptive validity is the measure by which findings are described accurately and objectively. To mitigate factors affecting descriptive validity, a data collection template was designed to support the record. The template guides the data extraction process objectively. Therefore, descriptive validity is considered to be consistent, and the factors are objectively controlled.

#### Theoretical validity

The theoretical validity is determined by the reviewer's ability to capture what is intended to be captured. In addition, confounding factors such as bias and reviewer selection play an important role.

Identification and Sampling: Studies may have been inadvertently omitted. To mitigate this, the search was supplemented with a retrospective sampling after reading the full text. Biases associated with reviewers may appear during selection and data extraction. Study selection was made by an individual author. To reduce these factors that compromise validity, study identification was assessed by creating a set of reference articles. This was done by the first author, through snowball sampling. A small set of new studies was obtained during this phase, indicating that the overall conclusions of this review will not change. There are other potential compromising factors, such as activities describing the study that may be omitted, misinterpreted, or incompletely reported. This may affect the classification of activities. As a control action, the first author checked the extraction.

Data extraction and classification: During this phase, the reviewer's bias is also a threat factor. In this phase, it is considered useful for one person to extract the information and another to review the extraction. To reduce this threat factor, the first reviewer evaluated all extractions performed by the professional. Although this step involves human judgment, the threat factor can be eliminated.

#### Interpretative validity

Interpretive validity is achieved when the conclusions are drawn are in accordance with the data, and therefore, adjust and reaffirm the conclusion validity. A threat factor in data interpretation is researcher bias. However, the authors have experience in similar reviews, which reduces the threat factor related to data interpretation.

#### Repeatability

Repeatability requires detailed information about the research process. For this reason, the process followed is described in detail, as well as measures to reduce the threat factors to validity. In a direct way, repeatability was favored by using the control measures adopted.

## Results

### Publications overview

#### Publications per Year

The first study was published by Kalyuga[74] in 1999. Between 1999 and 2022, a trend analysis of the average number of publications grouped into six-year intervals is conducted. 0.5 per year were published in the first period (1999–2004), 1.6 per year in the second period (2005–2010), 2.1 per year in the third period (2011–2016), and 4.5 per year in the fourth period (2017–2022). There is a definite upward trend in the number of publications, especially over the most recent period (2017–2022), when there has been a more than doubling of publications.

#### Studies aims/objectives

**Fig. 6:**
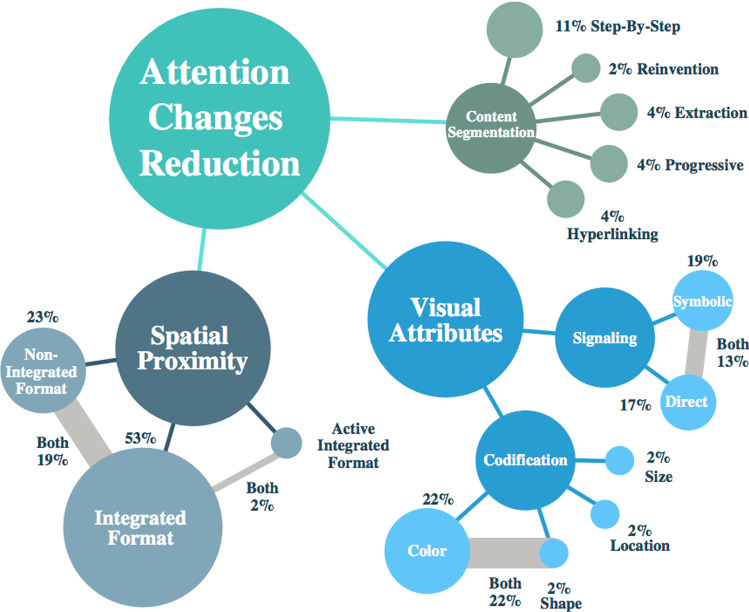
Strategies to attention changes reduction

Studies objectives identified were grouped in three groups, see Fig. 6:

- *Spatial Integration Techniques*: Comparative studies about instructional materials with physical integration formats 25% (n = 13). Integration by proximity [89]–[92], active [93] and non-integrated integration[94]–[97], to avoid attention changes was identified.
- *Comparative evaluation:* A set of studies 34% (n = 18) was as an objective the comparative evaluation between XR materials vs. paper-based instructions [58, 98, 99]. The evaluations were centered on cognitive load and user performance when attention changes occurred.
- *Visual Features assessment:* Several studies 42% (n = 22) using visual features in instructional material to deal with attention changes and reduce cognitive load [92, 95, 100, 101]. Visual features as color, shape and signaling were identified.

#### Field of instruction

**Fig. 7:**
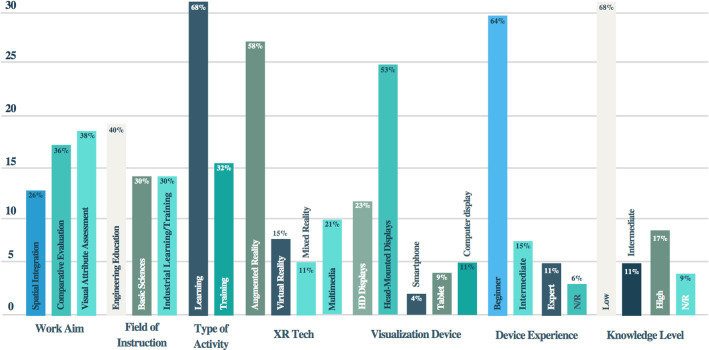
Summary of: studies aim, field of instruction, type of activity, xr technology, visualization device, device experience and knowledge level

The studies identified were aimed at the evaluations mentioned in Sect. 4.1.2. These evaluations were conducted in various fields of instruction. They were grouped into three main fields, see Fig. 7:

- *Engineering Education:* A set of studies (n = 22), equivalent to 42%, focus on engineering education. For instance, in higher education [96, 102, 103], electrical and mechanical engineering education[74], among others. The studies’ aim was to evaluate instruction material features in user cognitive load.
- *Basic Sciences*: (n = 17) studies equivalent to 32%, were applied in the educational area of basic sciences such as Biology [104], physics, chemistry [105], mathematics [75, 101] and health. For these studies the main objective was to define guidelines in the use of visual features for XR instructional material.
- *Industrial Learning-Training:* Finally, (n = 14) studies corresponding to 26% focused on learning, training and industrial assistance[106], mainly in assembly tasks [94], maintenance [92, 107] and reduction of errors during task execution [100].

#### Type of activity

The instructional material designed in the studies had several purposes as educational activities. The type of activities identified in the studies was:

- *Learning*: learning was identified as has the greatest number of studies (n = 37) equivalents to 70%. In other words, the material was used in a preliminary stage of training, during the presentation of information and knowledge learning.
- *Training*: 30% (n = 16) studies used the material in a later stage of training, in applications for military training [108], devices assembly[107], improving athlete performance [97] and in surgical procedure performance[89]. For example, Kotranza [89] studied real-time feedback for breast surgery, improving physicians' efficiency and psychomotor skills.

#### Technology

Several XR technologies were used in the studies reviewed. Augmented Reality, Virtual Reality and Mixed reality were used as educational tools. Next, the XR tech was used as a follow:

- *Augmented reality*: This tech was the most used XR technology with educational purposes. 55% of studies (n = 29) used Augmented Reality to deliver information to students and trainees, for instance, in engineering education and basic sciences[109]. AR in work stations [94, 110], class rooms [101], plant inspection and daily activities [111, 112].
- *Virtual Reality:* This technology was used in (n = 13) 25% studies for maintenance training and task assembly, scenario simulations and skill training. Mostly in work stations [58, 95] to evaluate the user performance using VR.
- *Mixed reality:* In (n = 5) 9% some studies MR was used for real object, tools or machine operation training. This tech was used because virtual objects can be manipulated in the scenario [103, 113].
- *Multimedia*: (n = 6) 11% of studies used Multimedia instruction to compare with XR technologies, mainly to evaluate spatial integrated formats [114, 115].

#### Visualization devices

Several devices were used in the studies experiments:

- *HMD*: Head-Mounted Displays or Headsets was the most used with 57% of studies (n = 30). Generally, in Mixed reality and Virtual Reality applications because of the ability to have the hands freed to execute a learning or training activity. Several devices were used in the experiments. AR devices as Epson-Moverio [97, 113, 116, 117], Google Glass [107, 112] and AR glass prototypes [91, 98, 101, 118], VR devices as Oculus Gear VR [37], Oculus Rift [101] and HTC[117], Mixed Reality Devices as Hololens2 [113, 119].
- *High Displays* or AR Projections. These devices were used in Augmented Reality and Virtual Reality 2D in (n = 12) studies 23%. For instance, [120–122] used high size displays to project AR content, for instance in Cooper [122], was used to visualize a car activity.
- *Computer Monitor:* (n = 5) 9% projects use AR in workstation using camera and desktop, e.g. [94, 96, 99], mostly to conduct assembly task training.
- *Tablets*: In maintenance activities (n = 4) 8% studies was use AR with tablets [100, 123], and learning content activities [109].
- *Smartphones*: AR is very common in smartphones. However, only a few studies (n = 2) 4% use AR in smartphone [92, 96].

#### Device experience level

The reviewed studies conducted assessment with participants whose level of experience in using these types of devices could be established as follows:

- *Beginner*: In (n = 33) studies, equivalent to 62%, subjects whose level of experience was Beginner participated. This was mainly observed with HMD devices for AR [97, 107, 112, 113] and VR [58, 95] in learning and training activities.
- *Intermediate*: (n = 9) 17% of the studies had participants with an intermediate level of experience. These studies mainly assessed those who used Smartphones [92, 96] or tablets [100, 109, 123].
- *Expert*: Five (n = 5) studies with expert participants comparatively evaluated the experience of novices Vs experts or were studies that only sought to capture the experience of the participant.
- *N/R:* Three (n = 6) 11% studies did not report the level of experience in using the device.

#### User knowledge level

The studies reviewed focused on learning and training activities. Its objective was to extend knowledge level and improve participants’ performance. Most of the studies assessed the level of knowledge of the participants in the tests. The reported level was as follows:

- *Low*: In 64% (n = 34) of studies were conducted with participants who reported low or no knowledge in the area of learning/training [58, 103, 107].
- *Intermediate*: In 11% (n = 6) the level of knowledge reported was intermediate. Mainly, in studies that seek to increase the performance of activities using XR.
- *High*: In (n = 8) studies, equivalent to 15%, the level of knowledge reported was expert. These studies focused on comparing the level of performance once the technology was used for training [89, 100, 123].
- *N/R*: (n = 5) studies did not report the users’ knowledge level.

However, studies such as Choi [96] and Fujimoto [98], where they used groups of subjects combining the level of knowledge and experience in the use of the visualization platform, stand out.

#### Graphical user interface features

The studies reviewed applied several visual features to avoid user attention changes. 58% of studies used features based on closeness between elements, while 17% did not use these features. Two formats related to proximity were identified: integrated format and active integration format. 17% of studies identified comparatively evaluated participant performance using the integrated and non-integrated formats. Finally, 40% of studies that applied physical integration complemented with other features. Task-time, accuracy and number of errors were used as performance measurement; and Learning, retention and knowledge transfer were used as learning outcome measurement.

Most studies focused on comparing factors that prevent attention changes, evaluating the user's performance and learning outcomes in several visualization platforms while performing learning or training tasks. In 17% of these studies, non-integrated formats were compared with integrated formats. In 28% XR technology (AR/VR/MR/Multimedia) was compared with printed material for instruction and training. In Table 3, a general description is shown. Another group of features identified are related to visual features such as color, shape, or symbols which are used to drive attention. The following point (4.2.1) describes how the features were used to avoid attention shifts in training tasks that use multimedia XR technologies.

**Table 3:** List of studies reviewed

Citation key	Ref	XR Tech	Spatial integration			Visual attributes		Segmentation	Hyperlinking	User knowledge	Effectt in user	
			Non-integrated format	Integrated format	Active integration format	Codification	Signaling				Cognitive load	Performance
Kalyuga, 1999	[18]	VR	√			Color				Low	↓	
Baudisch, 2003	[124]	AR	√				Direct (Circle), Symbolic (Halos)			Low		↓ (Time)
Seufert,2006	[105]	VR	√	√				Hyperlinking	√	Intermediate	↓	↓ (Time)
Jerome, 2006	[108]	AR		√		Size	Direct			Low	↑	↑ (Learning)
Bodemer, 2004	[93]	Multimedia		√	√			Step-By-Step		Low	↓	
Biocca, 2006	[125]	AR					Direct (Tunnel), Symbolic			Low	↓	
Wachsmuth, 2007	[90]	AR		√		Shape	Direct (Boxes)			Low		↓ (Time)
Ozcelik, 2009	[126]	Multimedia		√		Color				Low		↑ (Learning)
Fecteau, 2009	[127]	Multimedia		√		Position				Low		↑ (Learning)
Holsanova, 2009	[114]	Multimedia		√						Low		↓ (Time)
Cierniak, 2009	[128]	Multimedia	√	√		Color				Low	⇋	
Florax, 2010	[102]	Multimedia	√	√				Progressive		Low		↑ (Retention)
Schmidt-Weigand, 2010	[129]	Multimedia	√	√		Color				Low		↑ (Transfer)
DeCrescenzio,2011	[91]	AR		√			Direct (Boxes), Symbolic(Arrows)	Step-By-Step		Expert	↓	↑
Polys,2011	[104]	VR		√						Low		↓
Kim,2011	[130]	Multimedia		√						Low		↑ (Learning)
Kotranza, 2012	[89]	MR		√		Color, Shape	Symbolic(arrows)			Low		↑ (Learning)
Fujimoto, 2012	[98]	AR	√	√		Color, Shape				Low		↑ (Accuracy)
Herrema, 2013	[94]	AR	√	√		Color	Direct (Boxes), Symbolic(arrows)	Step-By-Step		Low		↓ (Time)
Webel, 2013	[123]	AR		√			Direct (Circle), Symbolic(arrows)	Step-By-Step		Expert		↓ (Time)
Kishishita, 2014	[75]	AR		√			Direct (Labels)			Low	↓	↓ (Time)
Hořejší, 2015	[99]	AR	√	√		Color	Symbolic(arrows)			Intermeadiate		↓ (Time)
Gavish,2015	[58]	VR	√	√		Color, Shape		Discovery-Hyperlinking	√	Expert		↑ (Time) ↓ (Errors)
Radkowski,2015	[106]	AR	√	√		Color	Direct (Boxes), Symbolic(arrows)	Step-By-Step				↓ (Time)
Madsen, 2016	[109]	AR		√			Direct (Labels)			Low		↓ (Time)
Kim, 2017	[97]	AR	√			Color, Shape	Symbolic(arrows)			Low		↓ (Time)
Rijnsburger,2016	[112]	AR		√				Extraction		Low		↑ (Learning)
Renner, 2017	[117]	VR		√			Symbolic(arrows)	Progressive		Low		↓ (Time)
Choi, 2018	[96]	AR	√			Color	Symbolic(arrows)			Low		↓ (Time)
Sirakaya, 2018	[92]	AR		√		Color, Shape				Low		↑
Polvi,2018	[100]	AR		√		Color, Shape	Symbolic(arrows)			Low	↓	↓ (Time) ↓ (Errors)
Bork, 2018	[119]	MR	√			Color				Low	↓	↓ (Time)
Azimi, 2018	[113]		√				Symbolic(Dots)				↓	↑ (Time)
Izard,2018	[37]	VR	√			Color	Direct (Contour)			Low		↑ (Accuracy)
Volmer, 2018	[131]	AR		√			Symbolic(arrows)			Low	↓	
Rao, 2019	[95]	VR	√			Color, Shape				Low	↑	↑ (Time) ↑ (Accuracy)
Kruijff,2019	[101]	AR	√	√		Color, Shape	Direct (Movements)			Low	↓	
Wang, 2019	[107]	AR	√			Color, Shape	Direct (Boxes, Circles)			Low	↑	↑ (Time)
Park, 2019	[103]	MR		√			Direct (Contour)			Low		↓ (Time)
Gabbard, 2019	[118]	AR		√				Extraction		Low	↓	↓ (Time)
Eiberg, 2019	[116]	AR		√						Low	↓	↓ (Time) ↓ (Errors)
Condino, 2019	[132]	MR		√						Low	↓	
Lei,201,922	[111]	AR	√							Low		↓
Makransky,2019	[115]	Multimedia		√							↓	↑ (Learning)
Cammerat,2020	[133]	Multimedia		√							⇋	↓ (Time)
Bang, 2020	[134]	AR		√							↓	
Markov-Vetter, 2020	[135]	AR		√			Symbolic(arrows)			Low	↓	
Lee, 2021	[136]	VR		√		Color	Symbolic (arrows)			Intermeadiate		⇋ (Time)
Vogt, 2021	[137]	VR		√			Direct (Label)			Intermeadiate		↑ (Learning)
Harada, 2021	[138]	VR	√				Symbolic (Radiation)			Low	↓	↓ (Time)
Cooper, 2021	[122]	VR		√		Color				Low	⇋	↓ (Time) ↓ (Errors)
Thees, 2022	[139]	AR		√						Intermeadiate	⇋	↓ (Time)
Krüger, 2022	[140]	AR		√						Low	⇋	⇋ (Learning)

AR: Augmented Reality, VR: Virtual Reality, MR: Mixed Reality, ↑: Increase, ↓: Decrease, ⇋ : No Effect, Time: Task-time, Learning:, Transfer: Knowledge Transfer, Accuracy: Task Accuracy, Error: Task execution error

#### Regarding spatial proximity

Three groups were identified related to spatial proximity between information source and spatial objects: a) Non-integrated, split, or distant format. Corresponds to formats whose design is spatially distant, that is, with a spatial separation that avoids spatial integration. b) Integrated format, defined by Schroeder [76], as formats whose information sources are close to each other. c) Active Integration Formats are used when trainees receive information in a non-integrated format and must perform the integration themselves by interacting with the content.

**Fig. 8:**
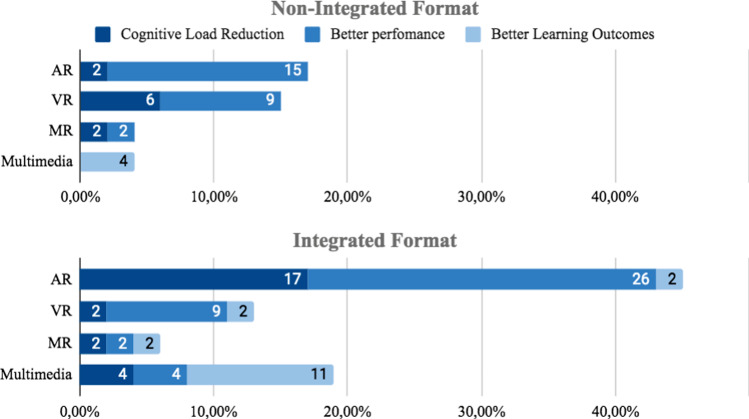
Effect in user of non-integrated and integrated format

- *Non-integrated formats:* Printed instructional materials are a kind of non-integrated format, as it requires a constant shift of attention between the guidance provided by the material and the actual setting where the task is performed. We identified (n = 20) 38% studies that used this type of format, 21% (n = 11) used only non-integrated format. In AR, 19% (n = 10) of the 53 studies reviewed use this format, 2% (n = 1) gets cognitive load reduction, 11% (n = 6)) better task-time, 2% (n = 1) better accuracy and 2% (n = 1) better overall performance and 2% (n = 1) did not report reduction in cognitive load or performance. Of this 21% (n = 11) that used non-integrated format, only two studies were evaluated regarding cognitive load. Azimi [113] and Kruijff [101] reported low cognitive load in trainees with low knowledge level. However, Wang [107] gets opposite results and reported high cognitive load. Complementally, in the use of non-integrated format, 28% (n = 15) of studies used codification features and 21% (n = 11) used signaling ones. In VR, 11% (n = 6) used Non-Integrated format. In Kalyuga [74] and Seufert [105], which comprise 6% (n = 3) of studies reviewed, reported low cognitive load, but in contrast, Rao [95] reported high cognitive load. In regard to performance, 4% (n = 2) reported short task-time completion and 9% (n = 5) showed better overall performance. In Multimedia 6% (n = 3) of studies used this format, 4% (n = 2) reported better learning outcomes and there were no reports on cognitive load or performance. Finally, in 19% (n = 10) of studies, apart from the non-integrated format use they were conducted also using codification or signaling features.
- *Integrated Formats:* 72% (n = 38) of the studies selected used integrated formats, see Fig. 8. 58% (n = 31) of all studies reviewed used only integrated format, 32% (n = 17) used integrated format along with visual features and 19% (n = 10) compared Integrated format with non-integrated formats. In AR 38% (n = 20) of all 53 studies used this format and 19% (n = 10) found a reduction in cognitive load, 21% (n = 11) shorter task-time completion, 7% (n = 4) better learning outcome and 4%(n = 2)) reported better learning outcomes. In Multimedia, 19% (n = 10) applied integrated formats, 11% (n = 6) reported better learning outcome and 4% (n = 2) shorter task-time completion. In VR, 13% (n = 5) of studies use integrated format, 2% (n = 1) found a cognitive load reduction and 7% (n = 4) shorter task-time completion. In MR, 6% (n = 3) of studies used integrated format, 2% (n = 1) reported cognitive load reduction and 4% (n = 2) better performance.

*Active integration format:* Active integration requires trainees to perform an action to move content to an integrated position. Bodemer [93] compared active integration and static integrated formats where the trainee gets up to 75% better performance when active integration is used. Also, it was identified that active integration showed significant performance improvements compared to the non-integrated formats. In another study, Bodemer [141], reports the difference between 2D formats (Non-integrated, integrated and actively integrated), and identified the usefulness of active integration, especially in instructional materials with high difficulty and complexity.

#### Effect conditions identified

Some additional conditions to proximity effect were identified: Presentation format, complexity task and device Field of view. The influences of representation formats were identified: text-text (T-T), text-graphic (T- G), graphic-text (G-T). Trainees who used text-text (T-T) and text-graphic (T-G) representation formats had better performance results, in contrast to graphic-text formats, who had lower performance results. Finally, some studies propose a design guideline based on task-type.

**Fig. 9:**
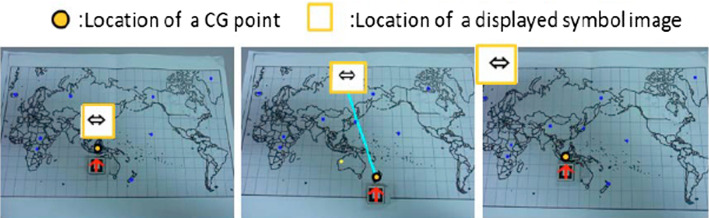
Fujimoto Study [98]. On the left, Integration by proximity: the information is displayed close to the object. On the center: information is displayed in a random position with respect to the object. On the Right: Non-integrated, where Information is displayed in a position far from the object

In the selected studies, the integrated format demonstrated its advantages compared to the non-integrated format. The results obtained improvements in performance, task time, precision, efficiency and, to a lesser extent, learning. Likewise, in the eyetracking analysis, the integrated format generated more integrative saccades. This explains that the integrated format encourages the use of a processing pattern, and the user process the information as a single unit, while when they are not integrated, they process them as two different units (Fig. 9).

#### Regarding visual features

**Fig. 10:**
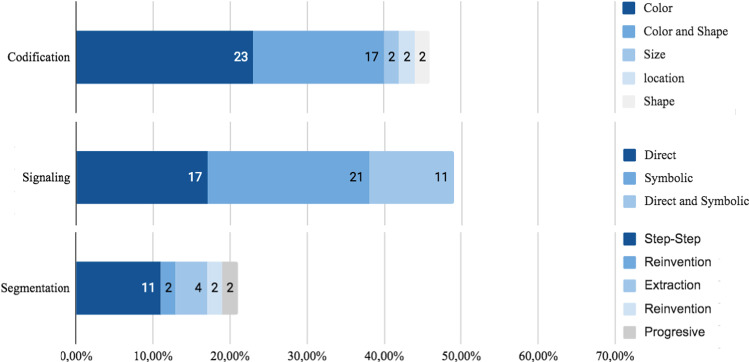
Visual features

Several visual features were used to reduce cognitive load and improve performance. In Fig. 10, a summary of visual features is shown.

##### Codification

The main use of codification was to provide correlated information. Therefore: color 40% (n = 21), shape 19% (n = 10), size 2% (n = 1), and positioning 2% (n = 1) were used as codification features. 44% (n = 23) of all studies used at least one codification characteristic, as shown in Table 3. The most frequent use of color was to apply the same color tone for content with correlated information source. A strategy aimed at reducing information pinpoint as well as cognitive load. Although, 55% (n = 29) of studies did not use any codification feature. In AR, 23% (n = 12) of all studies used codification, 4% (n = 2) reported a cognitive load reduction, 13% (n = 7) reported shorter task-time completion, 6% (n = 3) better performance results and 2% (n = 1) better learning outcome. In VR, 11% (n = 6) use codification features, 2% (n = 1) reported reduction and 2% (n = 1) no reduction in cognitive load, 4% (n = 2) better accuracy results and 4% (n = 2) a decrease in the number of errors. However, 4% (n = 2) reported the longest task-time completion. In MR, 2% (n = 1) reported a low cognitive load, 2% (n = 1) shorter task-time completion and 2% (n = 1) better learning outcome. Finally, in Multimedia, 6% (n = 3) reported better learning outcome but no performance result was reported, see Fig. 10. Shape was used as codification feature always complementing color codification in 17% of studies, 11% (n = 6) in AR, 4% (n = 2) in VR and 2%(n = 1) in MR. Generally, Color as the visual attribute mostly used.

In Rao [95] used color and shape to indicate the instruction location that the trainee should follow; this use was associated with lower mental effort in trainees. Kim [142] showed that color coding improves information comprehension. Related to time-task completion, in Sirakaya, there was no evidence of shorter task-time completion associated with color use [68]. Therefore, Sirakaya [92] mentions the need to limit the number of colors used, since the simultaneous use of many colors could eliminate any positive effects [92] and recommends a maximum use of 5 colors simultaneously. In Kruijff [92, 101] the color preference on labels was identified, its influence on text readability and its correlation with the background. Blue was the most preferred by test participants with OST (optical see-through) devices, gray and green were the preferred for VST (video see-through) devices. The impact of color use was evidenced by eye-tracking analysis, where it was observed an improvement on retention and learning results. In studies by Ozcelik [126] and Choi [96], it was observed that the average duration of ocular fixation indicated deeper processing. Thus, trainees who used color-coded format, paid more attention to the color-coded information in contrast to trainees who received un-coded information. It was also evident in Herrema [94], that the use of the same color to code the instruction (in text) and signaling (using some figure), its positioning in the workspace where the action must be performed improved task-time completion. In the Rao [95], Choi [96] and DeCrescenzio [91] studies, the color was used to code the text describing the task objective, 3D models objects and visual signals associated with the final objective. In other studies, such as Fujimoto [98] and Gavish [143], color coding was used, which complemented with graphic elements, such as boxes, indicators, icons and arrows [89, 97] also allowed for an improvement and shortening of tasks.

##### Signaling

**Fig. 11:**
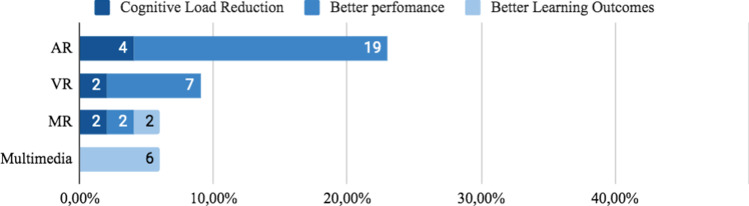
Effect in user with codification features

Van Gog [78] defines signaling as the set of signals or clues used to guide attention to the relevant elements of the instructional material. Signaling has been used in several ways to reduce cognitive load and better performance as well, see Fig. 8. In 28% (n = 15) of studies, direct signaling techniques were used. The most used characteristics were, labeling with 6% (n = 3) and boxes with 9% (n = 5). Also, 21% (n = 11) of studies used symbolic signaling techniques to indicate the target position required, 17% (n = 9) used arrows and 2% (n = 1) used a dot. Finally, 21% (n = 11) studies used other target positioning signals such as transparencies, flashing dots, highlighting tool, and animation. The most used characteristic was transparency (with 13%), used to highlight areas or elements of interest where attention is desired. Also, flashes, highlights or blinks, used color or other features. Kruijff [101] evaluates vertical, horizontal, and rotational movements getting cognitive load reduction and drive the user’s attention (Fig. 11).

**Fig. 12:**
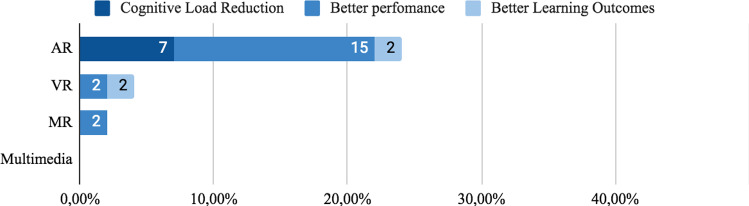
User effect when using direct signaling features

*Direct Signaling:* As a common strategy, primitive geometric figures such as squares, rectangles, and circles were used to frame the object of interest[113]. Direct signaling was the most used feature in AR Material, where 23% (n = 12) studies reported their used, 13% (n = 7) cognitive load reduction, 13% (n = 7) better performance results and 2%(n = 1) better learning outcomes. In VR and MX, only 2% (n = 1) of studies report better performance results and 2% (n = 1) better learning outcomes, see Fig. 12.

In Herrema[94], Wachsmuth [52, 90], DeCrescenzio [91], Kishishita [75], Wang[107], used boxes to limit objects and guide their identification within the visual field. However, some studies used primitive shapes with segmented contours as in Park [103] and Izard [37], where object detection achieved better performances results. Bodemer [93] used labeling to direct attention within inner and outer field of vision. Bell [144] used to connect lines to establish a visual link between the object of interest and its label. In Tatzgern[145] and Madsen [109], it states that, for a better effectiveness labels must be perpendicular to user view, the pinpoint located in its center, the connection line should not occlude the object but as short as possible to maintain a compact display. The use of labels for comparison tasks should reduce the use of connecting elements (preferably lines) and not reduce the field of view. For search tasks, the field of view should be expanded using connecting lines with greater contours and evident [104]. The labeling was also accompanied by subtle animation. In Kruijff [101] lateral, vertical and circular animations were given to labels. Circular animation was mostly identified in VST (video see-through) devices. For VST (video see-through) devices, blinking obtained better results. The combination of direct signaling with animations was also observed in this study. Biocca[146] uses a guide based on a flexible tunnel of frames that begins at the user's head and ends at the object location and orientation, while showing an animation that progressively reduces the frame size till it reaches said object. Circles were also used in Baudisch [124] and Wang [107], to surround target objects out of field of view with rings that reach its limits indicating the presence of more objects. This technique was used to indicate location and infer the distance to target based on the ring arc size. A variation of this technique is proposed by Renner [117], where circles in waves form propagate towards the target, like the concentric circles that appear when throwing a stone in the lake, only with a reverse direction. These feature combinations show error levels similar to techniques that use arrows. In addition, there was a significant reduction in task-time completion. However, these techniques have limitations with target positioning since its representation is difficult outside the user's field of view.

**Fig. 13:**
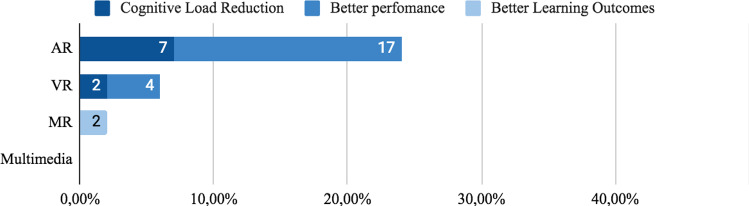
User effect in user when using symbol signaling features

*Symbol Signaling:* Some studies use symbols to guide users’ attention. Symbol signaling was the most used feature in AR, where 9% (n = 5) of studies reported cognitive load reduction, 11% (n = 6) reported better user performance and 2% (n = 1) better learning outcomes. In VR, only 2% (n = 1) of studies reported cognitive load reduction and 4% (n = 2) better user performance. In MX, only 2% (n = 1) reported better learning outcome. In Multimedia none of the 53 studies used symbols to drive users’ attention, as seen in Fig. 13.

For instance, Hořejší [99] used arrows, Biocca used omnidirectional attention tunnels [125, 147] and Baudisch [124] used Halos. In Renner [117] three techniques were compared to locate objects outside the field of view (like arrows, waves, and radar bleeping). Attention diversion using arrows showed better results in task-time completion, precision actioning, information readability, and technique usefulness. A technique variant is to place an arrow on the screen outline pointing towards the target location. As a complementary feature, animated 3D arrows were used in Radkowski [110], Choi [96] y DeCrescenzio [91], to indicate turns and point towards parts to be assembled. This combined use reduced the task-time completion as well as the number of errors made. Another identified combination was predictive signals, where the principle of predictability is applied to indicate the location where the visual stimulus will appear. Using arrow prediction, it was possible to increase task performance and considered highly suitable for AR environments, according to Volmer[131], due to mental effort reduction results.

#### Regarding content segmentation

##### Segmentation

**Fig. 14:**
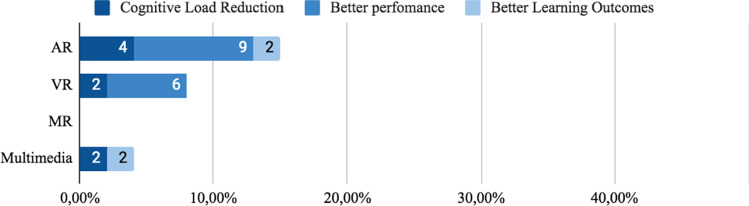
User effect when using Segmentation techniques

In some studies, content required to execute a task exceeds the available field of view space and could overload the trainee, see Fig. 14. In 21% (n = 11) of studies, content segmentation was divided into smaller parts to avoid or reduce attention changes. In 9% (n = 5) of studies content was segmented with step-by-step instructions. Other studies used the discovery, proposed by Gavish [58], to keep content hidden, but easily found by clicking on an icon.

In AR, 9% (n = 5) of studies reported better user performance when using segmentation techniques, a cognitive load reduction in 4% (n = 2) and better learning outcomes in 2% (n = 1) of them. In VR, out of all 53 studies only 6% (n = 3) of them reported better user performance and 2% (n = 1) reported cognitive load reduction, any of them reported better learning outcomes. In Multimedia, only 2% (n = 1) of studies reported cognitive load reduction and 2% (n = 1) better learning outcomes. Finally, in MX no study reported segmentation techniques.

Segmentation techniques was the most used in AR, for example, Rijnsburger [112], proposes that labels contain a five word maximum to avoid mental overload. Progressive disclosure is also a segmentation technique used in VR environments, Herrema [94] applied it in interfaces overloaded with information to reduce mental effort. Discovery has been used in projects like SKILLS [58], Hanley [148] proposes this concept, which consists of placing an icon to be interacted with to temporarily display information. Liarokapis [121] and Gavish [58] uses labels to link information to objects in AR environments, however, some difficulties when handling detailed descriptions, due to the descriptive text length. Thus, it was divided into smaller sections controlled by the user through a scroll bar. Finally, another segmentation variant is information extraction. Gabbard proposes that irrelevant information be eliminated and that correlated information be grouped together [118]. Rijnsburger [112] states another variant as information segments through personalization, especially for devices with reduced field of view as well as using labels with a 5 word maximum.

##### Hyperlinking

This technique is based on relational hyperlink use where links use visual indicators to relate content and allow the trainee to control playback. Furthermore, in some measure, this technique was found to be more effective than an integrated format. For example, Seufert [105], evaluated learning material in molecular biology under this concept. Relevant words, images, or formulas were accompanied by hyperlinks. When the trainee clicks any of them, it points to the corresponding element in the presentation. The results showed a reduction in trainee cognitive load. Gavish [58], also implemented a similar technique to show details of an assembly procedure instructions done step by step.

## Discussion and conclusion

In the review process, it was identified that only a few studies focused on cognitive load evaluation. As Ens [24] suggests, AR designers need cognitive effects information to design more effective learning and training environments. The most recent studies are focused on user performance while learning and training, but only a few studies are focused on cognitive effects. XR technologies are most used for learning than for training. In contrast to Gopher [149] state that AR is useful as a training technology strategy due to shorter task-time completion achieved by trainees. AR is the most used technology, mainly using Headsets as display devices. According to Zhang [150], being hands-free it allows to manipulate objects and tools.

XR technologies are used by novice and entry-level users, thus, as Gutierrez and Agati [55, 151] states, XR technologies are useful in learning/training preliminary phases for entry-level knowledge trainees.

## Regarding spatial proximity

Three spatial formats were identified: Non-integrated, integrated and Active Integration format. The Non-Integrated format allowed for trainees to achieve the best results by using AR technology, mainly, better user performance (task-time completion) and Cognitive load reduction. In the review, it was found that non-integrated format represented better learning outcomes when used in Multimedia technology. Generally, Non-Integrated format achieved a small cognitive load reduction in all XR technologies and better user performance but not better learning outcomes. In contrast, as Gloria Mark [152] states: trainees often compensate spatial separation between information sources by executing tasks faster. This justifies the users’ higher performance but also induces a higher overall cognitive load, higher workload, stress, high level frustration and more pressed for time. Due to these harsh training conditions, it is possible that the learning environment was inadequate for knowledge transfer and retention.

Several studies are based on close proximity stimuli causing associations between said stimuli, as mentioned by Ozceli [126]. Kalyuga [74] supports this thesis, adding that the distance between integrated formats should be the minimum possible. However, the distance or separation measurement unit is not specified. In the review process, no study evidenced the establishment of any rule relating the separation distance with any of cognitive load measures. In this sense, Ogueta [153] specifies this separation variable in angles measured in degrees, it is also identified a proportional correlation between distance, task-time completion, and number of errors made. In other words, the greater the separation distance, the longer was the time for task completion, and higher number of errors made. In addition, in XR technologies the visual separation uses a 3D coordinate system. This implies that separation could not be on an angle, it should be in spatial metrics as inches or centimeters since it must be coordinated with workspace real-world measurements. Additionally, the coordinate system implies movement, therefore it could cause depth perception issues, as Ping [154] describes. To solve this limitation, Diaz [155], proposed guidelines to design user perception techniques and take advantage of users' visual abilities to better blend the physical and virtual world.

The Integrated format was widely used in AR, reporting reduction in cognitive load, better user performance and better learning outcomes. However, in more recent studies, the results related to the reduction of cognitive load are not conclusive [122, 139, 140]. Possibly due to a better understanding and use of technology. However, the best results in learning outcomes were achieved in multimedia technology. XR Integrated format achieved positive results in relation to cognitive load reduction and lower mental effort. This could be due to dual information source processing, according to Fujimoto [98]. In integrated formats, information sources are processed as a single information source, i.e. when the object and source information are close to each other, the user processes all the information at the same time reducing the mental effort. Despite this result, on some studies as Radkowsky[106] and Sirakaya [92], the task-time completion advantage is little or non-significative. This result could be due to the little experience with this devices as Seufert mentions; or also entry-level knowledge and a small trainee sample as Sirakaya [92] reports. Regarding learning outcomes, as Holsanova [114] and Mayer[156] states, spatial contiguity is decisive to facilitate learning. However, in XR technologies we found more prominent results in Cognitive load reduction and user performance than learning outcomes. In learning outcome, the results could be influenced by test design. Seufert and Sirakaya used ad-hoc test, and as Makransky [115] mentions, the subjective measures can provide different information to test the theoretical mechanisms involved in multimedia learning. In XR technologies, Non-integrated and Integrated formats frequently were accompanied by visual features (codification and signaling), complementing the effect to obtain better learning or performance results.

As Mayer [71] states, spatial contiguity principle has a strong effect when the following conditions are evidenced: Low knowledge level, Non-Redundant text and imagens, complex learning sessions or interactive formats. In the review it was not possible to identify these conditions in all 47 studies. However, we can stablish that trainee knowledge level influences task-time completion results when a spatially integrated format is used [75, 101]. Likewise, it was observed that performance increases for trainees whose previous knowledge on the subject is high. However, it showed that in most studies the participants had a low level of experience and low knowledge level. The foregoing shows that although good performance results can be obtained, these could be significantly improved if tests are carried out with participants with high prior subject knowledge [130]. Similarly, the effectiveness results could be justified, as mentioned by Ginns [157] and Polvi [100] influenced by the complexity and type of task, such as information search tasks and information comparison tasks.

Another condition identified was the representation format. The user's working memory behavior is not the same when different representation formats are evaluated. Generally, representation formats are grouped into symbolic (text and other symbols) and pictorial (images and other representations). Likewise, in this type of spatial integration format, the integrations between text-text and text-graphic representation formats showed better trainee behavior with respect to learning performance and comprehension. However, the studies that showed improvements in learning performance did not show significant differences in task-time completion or mental effort [93]. This is because symbol representations are easy to interpret or does not require experience for interpretation [158]. However, pictorial representations such as graphics require prior knowledge and visual spatial ability, to be adequately interpreted, which implies higher working memory processing [133]. Additionally, the representation format processing could be influenced by the its complexity, that is, low complexity representations such as 2D graphics require greater visual spatial ability and processing than the high complexity representations such as 3D models and photographs [157]. Finally, there were differences observed between visualization platforms. For the AR platform with Displays, an increase in the task-time completion was observed, which may show the presence of high cognitive load. For HMD, more precision was observed in the identification of information, but not task-time completion improvements. Finally, in training activities, the integrated format showed better user performance. However, for learning material is difficult to provide concrete design guidelines because when trying to use such guidelines mismatched results were obtained [159] and also due to integrated format results in learning not being conclusive, as Beege [159] reports. In the same way, Cammerat [133] states that knowledge transfer and retention results are mixed, therefore, proposes more studies be performed regarding spatial proximity and learning outcomes. Studies that have shown mixed results of the principle of spatial contiguity, such as Cammerat [133] and Beege [159], use objective evaluation such as eye-tracking and were done on multimedia technology. However, in this review, there was no evidence of any study that evaluated in depth the spatial contiguity principle with XR technologies for learning or instructional material design. Although some multimedia principles are replicable in 3D technologies as mentioned by Lacoche [160], the use conditions of multimedia technologies are different, due to the workspace size and visual aspects involved.

## Regarding visual attributes

The most used codification technique was color codification. The content of all related information sources uses the same color tone. This technique was the most used in AR technology, participants achieve better performance and a significant cognitive load reduction. This result is possible due to the easy mapping principle, which establishes the ease required to identify objects in a workspace, reported by Tatzgern [145]. In the same way, studies showed improvements in retention and knowledge transfer. These results were due to the efficiency in locating complementary information sources, and the ability to attract user attention to objects with highlighted information. The preference for blue in AR (OST) devices may originate because this color coincides with the cone sensitivity in the peripheral visual field. This indicates that the device display influences the color selection and may be related to the amount of light present in the projections of each device. According to Ozcelik [126] a 5 color maximum is recommended to be used simultaneously. These results are consistent with the Sirakaya research [92], where an adverse effect was noticed due to confusion by a high number of colors used simultaneously. Likewise, Kalyuga [74] also agrees when reporting that using too many colors simultaneously can impose a significant load on working memory and eliminate any positive effect of color codification. Finally, Kalyuga [74] proposed color codification as an alternative to spatial integration to reduce attention changes, especially in computer-based instructional material with few space available. Kaylyuga proposition is based on an eye-tracking study, when working memory processing using color coding format is similar to spatial integration format. However, some limitations such as the number of colors available to use simultaneously [126]; possible perception changes of color in HMD devices caused by lighting and the device's projection technology, according to Livingston [161]; and the colored element position, since color perception worked best the closer it is to central vision focal point and also requires the user’s gaze tracking to detect the best element positioning.

Direct signaling technique was the most used in multimedia technology. Primitive shapes, labels and subtle animations were used where overall results achieved better learning outcome. However, in AR better user performance was achieved. The shape and label uses are related to device Field of view, i.e. locating objects of interest inside or outside the field of view being possible due to task-type requirements. In other words, the label elements selection depends on task-type as mentioned by Polys [104]. As well, to Madsen [109], search tasks needed for visual exploration in the work field is proportional to visual field size. In other words, for large visual field devices, more visual exploration is required and therefore a greater cognitive load is demanded. However, according to Tatzgern [109] for comparison tasks, the visual field should be limited, and although there are continuous changes in attention focus, the location is already known and does not require additional spatial searches, which forces higher cognitive processing. Finally, for user performance training purposes the field of view should be constrained not widen, identified by Ren[162] where he correlates FOV size and performance.

Likewise, the effectiveness of vertical, horizontal and label rotation animation was established to drive attention. Therefore, the use of subtle animation in elements has some advantages. Mainly because it worked best at the visual field edge, while color and shape worked best the closer to central vision focal point it is [75]. This condition does not require user gaze tracking to identify the best element positioning and could be used in large field of view devices. Another advantage is represented by the use of animation of a single object at a time, this could be used to guide procedures and focus attention on the main object.

The signaling based on symbols preferred used arrows as elements for task-time completion and number of errors made reduction. Despite using rings or arcs to guide attention, arrows were the most used. This is possible due to the decrease in the processing required to interpret information represented by symbols. Herrema states when symbols are used they can be quickly interpreted by the user, since recognizing a symbol reduces the processing required for recognition[94]. However, it represents a significant limitation since known symbols must be used to avoid misinterpretation. Additionally, symbols must indicate the action, for example, the arrow indicates direction of movement or displacement [91]. This may represent a limitation related to the type of symbols that can be used, since they must meet recognition conditions.

## Regarding content segmentation

Instructional and training materials are becoming more comprehensive and complex. Due to this, the amount of content is greater. Therefore, content segmentation was identified as a technique to avoid cognitive overload in trainees. Despite being used in few studies, effects were identified in increasing the performance and reducing cognitive load. However, segmentation techniques represent a weakness evidenced by Hanley [148], since the required instructions are divided into several spaces, generating a violation of the spatial contiguity principle. However, under restrictions such as a 5-word maximum, adverse effects could be avoided. This is consistent with the principle of conciseness mentioned in ISO 9241–12[163], which suggests that users should not be overloaded with extraneous information. Also consistent with the results of Florax [102]that indicate that learning can be improved by segmentation to a greater extent and to a lesser extent with labeling.
